# An empirically driven data reduction method on the human 450K methylation array to remove tissue specific non-variable CpGs

**DOI:** 10.1186/s13148-017-0320-z

**Published:** 2017-02-02

**Authors:** Rachel D. Edgar, Meaghan J. Jones, Wendy P. Robinson, Michael S. Kobor

**Affiliations:** Department of Medical Genetics, BC Children’s Hospital, University of British Columbia, Vancouver, Canada

**Keywords:** Non-variable, 450K, Tissue, Filter, Power, Multiple-test correction, DNA methylation, Dimensionality reduction

## Abstract

**Background:**

Population based epigenetic association studies of disease and exposures are becoming more common with the availability of economical genome-wide technologies for interrogation of the methylome, such as the Illumina 450K Human Methylation Array (450K). Often, the expected small number of differentially methylated cytosine-guanine pairs (CpGs) in studies of the human methylome presents a statistical challenge, as the large number of CpGs measured on the 450K necessitates careful multiple test correction. While the 450K is a highly useful tool for population epigenetic studies, many of the CpGs tested are not variable and thus of limited information content in the context of the study and tissue. CpGs with observed lack of variability in the tissue under study could be removed to reduce the data dimensionality, limit the severity of multiple test correction and allow for improved detection of differential DNA methylation.

**Methods:**

Here, we performed a meta-analysis of 450K data from three commonly studied human tissues, namely blood (605 samples), buccal epithelial cells (121 samples) and placenta (157 samples). We developed lists of CpGs that are non-variable in each tissue.

**Results:**

These lists are surprisingly large (blood 114,204 CpGs, buccal epithelial cells 120,009 CpGs and placenta 101,367 CpGs) and thus will be valuable filters for epigenetic association studies, considerably reducing the dimensionality of the 450K and subsequently the multiple testing correction severity.

**Conclusions:**

We propose this empirically derived method for data reduction to allow for more power in detecting differential DNA methylation associated with exposures in studies on the human methylome.

**Electronic supplementary material:**

The online version of this article (doi:10.1186/s13148-017-0320-z) contains supplementary material, which is available to authorized users.

## Background

Population studies that interrogate epigenetic signatures associated with environmental variation and disease are becoming increasingly common. The challenge with the majority of epigenome wide association studies (EWAS) of environment and disease is that the epigenetic signals, in terms of detectable number of epigenetic changes and the effect size of changes, between groups are relatively small compared to those observed in EWAS of development, tissues or cancer. Therefore careful and specific methodological steps need to be implemented in analyses to separate any true biological signal from stochastic variation in DNA methylation (DNAm), a phenomenon commonly referred to as noise [1].

One of the most common types of population based epigenetic studies is the examination of DNAm using the Illumina Infinium 450K array (450K) or its related arrays [2]. The Illumina series of DNAm arrays, while highly useful as tools for epigenetic studies, were not designed for any specific human tissue, and a large number of cytosine-guanine pairs (CpGs) lack variability within single tissue studies on the arrays [3–8]. CpGs that are non-variable in a study of a specific disease or tissue may be variable in another context and therefore are still valuable on the 450K. However, these tissue specific non-variable CpGs contribute to the high dimensionality of the 450k data and partially necessitate the need for severe multiple test correction. In an effort to rigorously determine the epigenetic signals of environmental exposure and/or disease phenotypes, dimensionality reduction techniques are often employed. These include mixture modelling, principal component analysis, weighted gene co-expression network analysis and elastic net models, among others [9–12]. While these techniques are effective for high-dimensional data reduction, they do not take into account the wealth of independent DNAm data available to build empirical data reduction filters. A common data-driven dimensionality reduction technique is to remove non-variable CpGs from within a specific study and then test only variable sites for association with the exposure of interest [3–8]. While this practice can reduce severe multiple test correction penalties, it can introduce a bias toward significant results [13]. A promising alternative from gene expression analyses is to use a filter based on prior biological knowledge from independent data, which can be highly effective in improving sensitivity while maintaining specificity [13].

Here, we have developed an empirically derived data reduction method in the form of CpG lists which are non-variable in independent cohorts of samples from three commonly used human tissues: blood, buccal epithelial cells and placenta. We anticipate these independently identified non-variable CpG lists will be useful for confirmation of a lack of variability at CpGs in 450K studies of interest. As such, our non-variable CpGs might serve as a benchmark to cross-reference CpGs also seen as non-variable in a study of interest so that these CpGs can be filtered prior to differential DNAm analysis. Removal of these independently verified non-variable CpGs should then allow for a reduced multiple testing space and allow for more power to detect differential DNAm in the study of interest. While this approach will be immediately useful for studies of 450K data, it will also provide a blueprint for similar approaches with emerging technologies such as the Illumina EPIC array. Our filtering approach for data reduction is focused on CpG-by-CpG EWAS analyses, which are very common approaches in DNAm analysis. However, this filtering approach also has the potential to improve the performance of other analyses where a strong signal is expected at a small subset of CpGs and noise in the data is a concern. In the context of the rapidly increasing number of DNAm datasets being produced, we have made our code available so that independent non-variable CpG lists can be rapidly developed for other tissues of interest on the 450K and the EPIC as data becomes available.

## Methods

### Data collection

The tissue datasets were collected from Gene Expression Omnibus (GEO) [14]. In all tissues, cancer samples were excluded, as cancer is associated with high DNAm variability [15]. For individual tissues, there were a range of exclusion terms by which samples were filtered (Additional file 1: Table S2). Exclusion terms were based on whether the term indicated cancerous tissue, a tissue other than the tissue of interest or a species other than human. In general, data was downloaded as non-normalized betas, but in some cases, M values were converted to beta, and normalized data was used. Each tissue dataset was then filtered down to the minimal number of CpGs with DNAm values across all samples of a tissue (blood 469,961 CpGs, buccal epithelial cells 420,374 CpGs and placenta 484,621 CpGs).

### Quality control

**Fig. 1:**
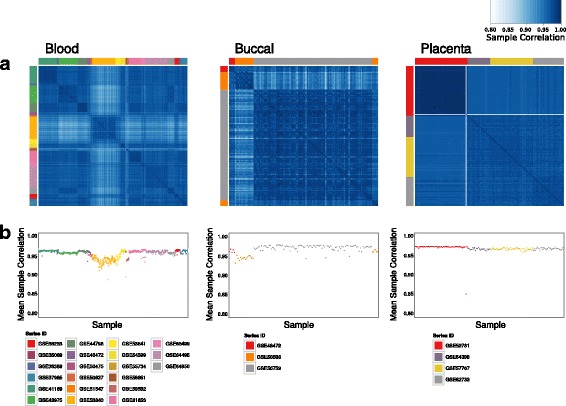
Quality control of samples from GEO for each tissue type. **a** Heat maps showing sample-sample correlation values. *Side colours* show the study ID of each sample, and samples are ordered by study ID. **b** Plots of the average sample-sample correlation for each sample to show possible outliers and studies with overall low average sample-sample correlation

To remove CpGs and samples that consistently did not perform well on the 450K, CpGs were filtered if greater than 5% of samples had fewer than three beads contributing to the signal across all samples from a tissue. Samples were removed if 2.5% of CpGs in a sample had fewer than three beads contributing to the signal. Samples were also removed if they had low sample-sample correlation compared to all other samples of a tissue. One sample was filtered from placenta and four entire studies were filtered from blood (total of 158 samples from blood; see Fig. 1; Additional file 1: Table S2). The final studies and samples included are listed in Additional file 1: Table S3.

### Non-variable calling

To designate a CpG as non-variable in a tissue, a threshold of 5% range in beta values (DNAm level ranging from 0 to 1) between the 10th and 90th percentile was used [16]. While effect sizes as small as 1% are used in EWAS [8, 17, 18], we used a slightly more stringent definition of change in beta of 5% as we are asking only that the population as a whole varies by at least 5% and are not testing an effect size between groups. CpGs with less than 5% reference range of beta values in a single tissue population were considered non-variable in that tissue.

### Genomic enrichment

To explore the genomic context of non-variable CpGs, all CpGs were associated with gene features using the annotation described previously [19] and with CpG island features as provided in the Illumina annotation [2]. The count of non-variable CpGs located in each gene feature (promoter, intragenic, 3 prime region and intergenic) and CpG island feature (island, north and south shore, north and south shelf, and no island association) were compared to the background counts of all CpGs measured, in each tissue. To compare the non-variable CpG counts to the background in each region, 1000 permutations of random CpG lists were used to calculate fold change values over the background [20].

### Application of data reduction method

To reproduce the published findings of AHRR DNA methylation changes associated with smoke exposure, a linear modelling approach was used on previously published data [21]. In short, DNAm values were normalized using BMIQ [22], and cell composition was normalized between blood samples [23, 24]. A linear model was run at all CpG sites and delta beta effect sizes were calculated between smokers and non-smokers in the full dataset of 111 blood samples. To simulate a study with reduced power, ten permutations of 24 random samples (12 smokers and 12 non-smokers) were selected and the same linear model was run at all CpGs. To test the data reduction method, the CpGs in the ten smaller cohorts were filtered to 374,945 variable CpGs by overlapping the CpGs that were non-variable in GSE53045 (264,578 CpGs non-variable at a reference range of 0.05) and the blood non-variable CpGs identified in the independent samples (114,204 CpGs described above). Then, the same linear model was run on only variable CpGs. CpGs were associated to genes as previously described [19].

## Results

### Tissues showed similar levels of non-variable CpGs

DNAm data from publicly available studies was collected for blood, buccal epithelial cells and placenta (21, 3 and 4 studies, respectively). Meta-analysis of samples for each of the tissues showed generally high correlations (70% of sample pairs correlated above 0.95). While there were some samples with higher within study correlations than across study correlations, the overall high correlation of cross study samples can be taken as evidence of the consistency of the 450K across research groups (Fig. 1). While four studies of blood were removed due to low correlation, no obvious explanation of the lack of correlation could be found in the available study characteristic information (Additional file 1: Table S2). The generally high concordance of the DNAm samples from the same tissue but different studies gives us confidence going forward in the appropriateness of comparing variability across studies. After quality control of the data, 605, 121 and 157 samples were used from blood, buccal epithelial cells and placenta, respectively.

**Fig. 2:**
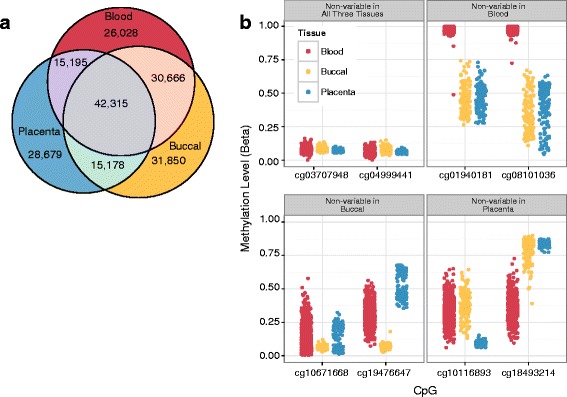
Non-variable CpGs had similar characteristics in all tissues. **a** Venn diagram showing the overlap of non-variable CpGs between tissues. **b** Methylation levels of representative non-variable CpGs from each tissue

A substantial number of tissue-specific non-variable CpGs were identified, thus providing a solid baseline for potential removal from studies of interest to reduce dimensionality. The total number of non-variable CpGs was similar across tissues: blood 114,204 (24%), buccal epithelial cells 120,009 (29%) and placenta 101,367 (21%) and showed a significant overlap of 42,315 non-variable CpGs (permutation *p* < 0.0001; Fig. 2a). Non-variable CpGs existed in either fully methylated or unmethylated state, with few non-variable CpGs observed at an intermediate DNAm level. In all tissues, the 99th percentile of non-variable CpGs had a mean DNAm greater than 0.80 or less than 0.16 (Fig. 2b). To test robustness of the non-variable CpG lists, we compared the list of non-variable CpGs prior to processing with a similar list generated after normalization or after cell type correction. We found that non-variable CpG lists overlapped by 90% with all processing strategies (Additional file 1).

**Fig. 3:**
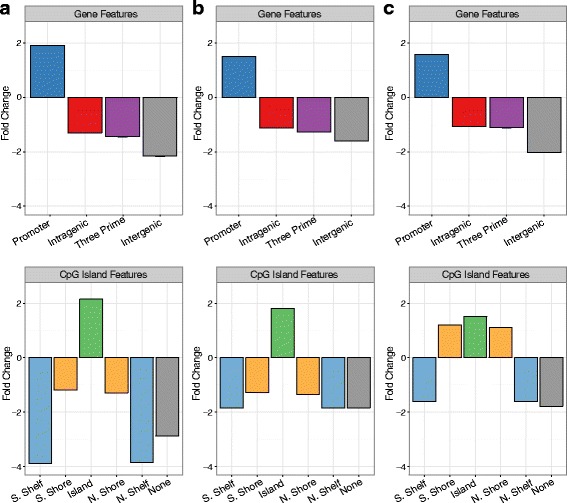
Non-variable CpGs were enriched in CpG island and promoters. All plots show the enrichment fold change of non-variable CpGs compared to all CpGs available for a tissue. Each pair of plots shows the fold changes in gene regions (*top*) and CpG resort features (*bottom*). **a** Blood non-variable CpGs. **b** Buccal epithelial cell non-variable CpGs. **c** Placenta non-variable CpGs

While exploring the biological role of non-variable CpGs that was not the primary focus of this analysis, we did observe that non-variable CpGs from each tissue were significantly enriched in promoters and CpG islands (relative enrichment = 2.46–8.20, false discovery rate (FDR) = 0.01; Fig. 3), with maximum enrichment in blood and lowest enrichment in placenta. Based on the large overlap in and similar genomic localization of non-variable CpGs between the three tissues, it is likely that the non-variable CpGs identified have similar underlying properties in each tissue.

### Application of data reduction method to smoking cohort

**Fig. 4:**
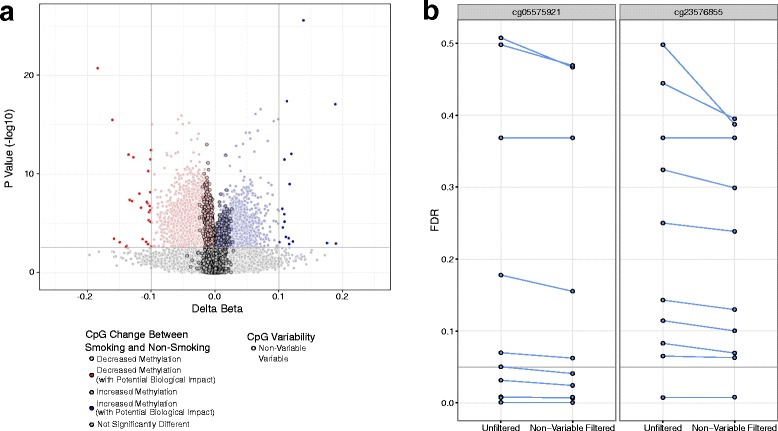
Multiple test corrected *p* values were lower in the filtered EWAS. **a** Volcano plot of the differential methylation analysis between smoking and non-smoking samples, with no filtering of non-variable CpGs. *Vertical lines* indicate a DNAm difference between 0.1. The *horizontal line* represents an FDR corrected *p* value of 0.05. Points are coloured to highlight CpGs exceeding both the biological and statistical cutoffs. *Points with a black outline* are CpGs found to be non-variable in blood. **b** Shown are the multiple test corrected *p* values (FDR) for the two CpGs of interest in AHRR. Lines connect FDR values between paired permutation sub-samples to show the trend between paired cohorts. The *horizontal line* shows the FDR values of 0.05

To test the utility of our filtering non-variable CpGs as a dimensionality reduction method, capable of improving statistical power and sensitivity, we attempted to demonstrate the gain in statistical power in reproducing a well-accepted true positive DNAm modification associated with smoking. In particular, one of the most reproducible biomarkers in DNAm association studies to date is decreased DNAm associated with smoke exposure at two CpGs in the gene body of AHRR [21, 25–28]. To validate our data reduction method, we used the AHRR signal in response to smoke exposure as a true positive. By reanalyzing all 111 blood samples available with smoking status in the original unfiltered data set (GSE53045) [21], we reproduced the finding of significantly decreased DNAm at two CpGs (cg05575921, cg23576855; FDR <0.05, delta beta 0.1) in AHRR. Interestingly, the non-variable CpGs often reached statistical significance (Fig. 4a), supporting that targeted removal of non-variable CpGs from EWAS improves specificity and reduces spurious associations.

To simulate a less powered study of smoke exposure, we randomly sampled the cohort down to 24 samples (12 smokers, 12 non-smokers) ten times. The same linear model, as used in the full cohort, was run on each of the ten randomly sampled smaller cohorts, but with either all 485,512 CpGs included in the EWAS or with filtering of 110,567 non-variable CpGs (filtered EWAS). This resulted in several interesting insights. First, in nine of the ten low powered EWAS sub samples, the multiple test corrected *p* values of the two true positive AHRR CpGs of interest were smaller in the filtered data set (Fig. 4b). Second, beyond AHRR, only six out of ten sub samples had any significantly differentially DNAm CpGs regardless of whether we used filtered or unfiltered data (FDR <0.05, delta beta 0.1). Third, in five of these six, the filtered data set EWAS resulted in more CpGs with significant differential DNAm. The greater significance of AHRR in the filtered EWAS suggested that filtration of non-variable CpGs should allow for prioritization of true positives, potentially even when the differential DNAm signal is not as strong as AHRR in smoking.

## Discussion

Here, we have developed an empirically derived dimensionality reduction method for EWAS, which can reduce noise in 450K data from tissue specific non-variable CpGs. Our proposed method for removing our empirically identified non-variable CpGs is to first confirm if they are also non-variable in the new dataset of interest and remove only those CpGs which are confirmed as non-variable, as presented in the analysis of the AHRR signal in response to smoke exposure. This procedure would avoid removing CpGs that were non-variable in the data collected previously, but do in fact vary in new data being analyzed from the tissue. Generally, previous analyses on 450K data have either filtered based on variability within the study data or not filtered the data on variability at all. We consider our filtration method to be a more moderate compromise between false positive and negatives. Our method is less biased toward false positives than filtering based on variability just in the study data, and also less likely to result in false negatives due to severe multiple test correction when no variability filter is to be used at all [13].

In defining our non-variable CpG list, we were agnostic to normalization methods and did not correct for batch effects between laboratories, beyond removing samples with low sample-sample correlations. We have therefore left in variability in the data due to technical factors that would have been minimized had we combined the data for normalization and performed batch correction. Our list of non-variable CpGs is thus conservative, but should be robust to study specific technical variability, increasing its utility in the community.

We have demonstrated the utility of the filtration in the analysis of smoke exposure in GSE53045, as the successful identification of differential DNAm at the true positive AHRR and the identification of more CpGs genome wide with significantly differentially DNAm. We do not propose simply observing more CpGs with differential DNAm as a good metric for the utility of our data reduction method, as some of the significant CpGs identified with filtration will be false positives. However, in combination with the observation of significant differential DNAm at the true positive, AHRR, more consistently with filtration, we are confident that our data reduction method will have utility in allowing identification of replicable differential DNAm in other datasets. Filtering for data reduction will be particularly useful when there is an expectation of CpGs with strong differential methylation signals (>5%); so, the expected magnitude of DNAm change should be carefully considered by the researcher before applying any data reduction. In concert with a stringent biological filter for the change in DNAm level between groups (5–10%) [1, 29], and validation of the 450K results with another technology such as pyrosequencing [1], this tissue specific DNAm data dimensionality reduction method may allow for better and more stringent identification of epigenetic signatures of exposure or disease.

## Conclusions

While the ability to define a tissue specific non-variable list will ultimately depend on the amount of data available for the tissue in public repositories, we expect there are already other tissues of interest with sufficient 450K data for which a useful list of non-variable CpGs could be developed. We have therefore made our code for building tissue specific non-variable lists available on GitHub (github.com/redgar598/Tissue_Nonvariable_450K_CpGs). We hope our analysis can be reapplied in the future to update the non-variable CpGs lists for blood, buccal epithelial cells and placenta as more samples become available, and be expanded to more tissues. Additionally, with the increased dimensionality of the newly released Illumina Infinium EPIC array, the need for tissue specific dimensionality reduction will be even greater. The analysis we have outlined and made available can easily be applied to EPIC array datasets as more are released [30].

## Additional file

Additional file 1: Table S1. Gene expression omnibus data description and additional analysis. Terms used to exclude samples not of interest in a given tissue. **Table S2.** Quality control filters for each tissue and the resulting final study, sample and CpG numbers. **Table S3.** Series IDs of the final samples used in the meta-analysis of tissue non-variable CpGs. Additional analysis on the stability of the non-variable CpG list with different data processing approaches. (PDF 63 kb)

## Abbreviations

450K: Illumina 450K human methylation array
CpG: Cytosine-guanine pair
DNAm: DNA methylation
EWAS: Epigenome wide association studies
FDR: False discovery rate
GEO: Gene expression omnibus
