# Supplementary material for: An empirically driven data reduction method on the human 450K methylation array to remove tissue specific non-variable CpGs
Source: Clin Epigenetics. 2017 Feb 2;9:11. doi: 10.1186/s13148-017-0320-z (PMC5290610; doi:10.1186/s13148-017-0320-z)
Supplement: Additional file 1: Table S1. — Gene expression omnibus data description and additional analysis. Terms used to exclude samples not of interest in a given tissue. Table S2. Quality control filters for each tissue and the resulting final study, sample and CpG numbers. Table S3. Series IDs of the final samples used in the meta-analysis of tissue non-variable CpGs. Additional analysis on the stability of the non-variable CpG list with different data processing approaches. (PDF 63 kb) [file 13148_2017_320_MOESM1_ESM.pdf]

Table 1: Series IDs of the final samples used in the meta-analysis of tissue non-variable CpGs.

Series	Samples	Tissue
GSE33233	19	Blood
GSE35069	6	Blood
GSE36369	20	Blood
GSE37965	30	Blood
GSE41169	95	Blood
GSE43975	96	Blood
GSE44798	50	Blood
GSE48472	11	Blood
GSE50475	5	Blood
GSE51547	1	Blood
GSE54399	24	Blood
GSE55734	6	Blood
GSE59592	6	Blood
GSE61653	64	Blood
GSE63499	23	Blood
GSE64495	113	Blood
GSE64950	36	Blood
GSE48472	5	Buccal
GSE50586	20	Buccal
GSE50759	96	Buccal
GSE52731	55	Placenta
GSE54399	24	Placenta
GSE57767	45	Placenta
GSE62733	33	Placenta

Table 2: Terms used to exclude samples not of interest in a given tissue.

Tissue	Exclusion Term
Blood	Brain,AML,leukemia,B-cell, gyrus,cortex,PBMC,CD19+, cerebellum,peripheral,cancer, marrow,troglodytes,vaginal, B cells,Peripheral,Cord,cord, Chimpanzee,paniscus,B cells, troglodytes,gorilla,Pongo, cerebellum,cortex,gyrus
Buccal	tumor
Placenta	Leukocyte

Table 3: Quality control filters for each tissue and the resulting final study, sample and CpG numbers.

	**Blood**			**Buccal**			**Placenta**		
**Data Processing Stage**	Studies	Samples	CpGs	Studies	Samples	CpGs	Studies	Samples	CpGs
Original Data Collection	21	883	469,987	3	121	420,381	4	158	485,512
After Removal of Samples with 2.5% NA across CpGs	21	763	469,987	3	121	420,381	4	158	485,512
After Removal of CpGs with 5% NA Across Samples	21	763	469,961	3	121	420,374	4	158	484,621
After Removal of Samples with Low Sample Correlation (Final Counts)	**17**	**605**	**469,961**	**3**	**121**	**420,374**	**4**	**157**	**484,621**

Non-variable CpGs Lists with Alternative Processing

In order to test how robust our non-variable CpG lists are to different data processing approaches, we re-ran our analysis on the buccal studies using the normalization methods BMIQ[22] and quantile normalization. Our buccal non-variable list was robust to normalization method. The non-variable CpG lists generated from BMIQ or quantile normalized data overlapped by 96% with our original non-variable list.

Additionally the effect of cell type correction was tested by first normalizing the cell compositions of all blood samples [23,24] and then defining the non-variable CpG list on the cell composition adjusted data. Here the non-variable CpG list calculated on cell composition adjusted and unadjusted data overlapped by 90%, implying our non-variable list is robust to data processing differences.
